# Supplementary material for: Genetic comparisons of interleukin-17 reveal a framework for complex signaling evolution
Source: bioRxiv. 2026 Apr 14:2026.04.13.718218. Preprint. [Version 1] doi: 10.64898/2026.04.13.718218 (PMC13105070; doi:10.64898/2026.04.13.718218)
Supplement: Supplement 3 [file media-3.zip › Supplemental files for paper/ERC/IL17xIL17_comparisons/IL17RE_IL17F.pdf]

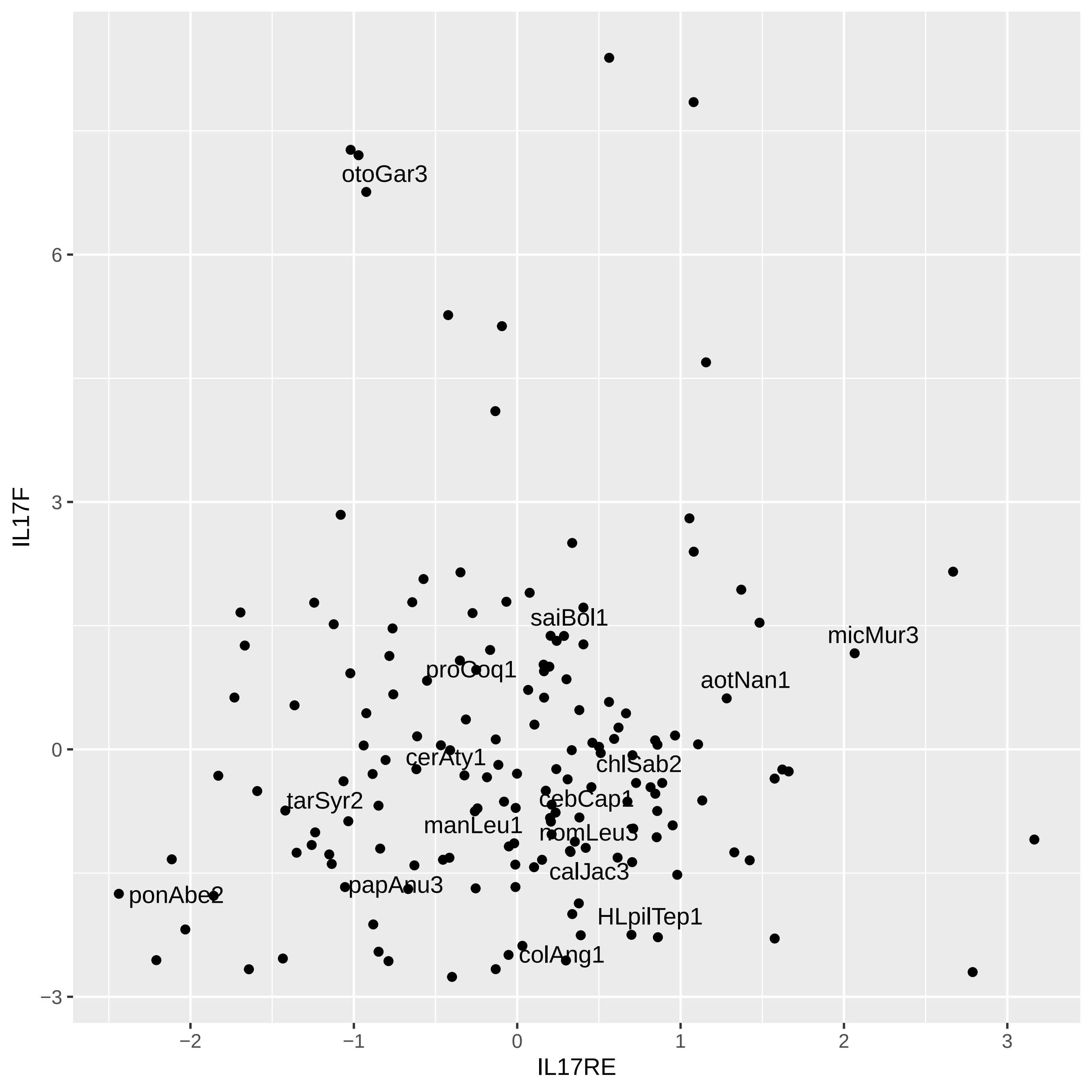

otoGar3
saiBol1
micMur3
proCoq1
aotNan1
cerAty1
chlSab2
tarSyr2
cebCap1
manLeu1
nomLeu3
calJac3
papAnu3
ponAbe2
HLpilTep1
colAng1
6
3
0
−3
IL17F
−2
−1
0
1
2
3
IL17RE
